# Genetic Evidence for Restricted Dispersal along Continuous Altitudinal Gradients in a Climate Change-Sensitive Mammal: The American Pika

**DOI:** 10.1371/journal.pone.0039077

**Published:** 2012-06-15

**Authors:** Philippe Henry, Zijian Sim, Michael A. Russello

**Affiliations:** Department of Biology, University of British Columbia, Okanagan campus, Kelowna, British Columbia, Canada; University of Bristol, United Kingdom

## Abstract

When faced with rapidly changing environments, wildlife species are left to adapt, disperse or disappear. Consequently, there is value in investigating the connectivity of populations of species inhabiting different environments in order to evaluate dispersal as a potential strategy for persistence in the face of climate change. Here, we begin to investigate the processes that shape genetic variation within American pika populations from the northern periphery of their range, the central Coast Mountains of British Columbia, Canada. At these latitudes, pikas inhabit sharp elevation gradients ranging from sea level to 1500 m, providing an excellent system for studying the effects of local environmental conditions on pika population genetic structure and gene flow. We found low levels of neutral genetic variation compared to previous studies from more southerly latitudes, consistent with the relatively recent post-glacial colonization of the study location. Moreover, significant levels of inbreeding and marked genetic structure were detected within and among sites. Although low levels of recent gene flow were revealed among elevations within a transect, potentially admixed individuals and first generation migrants were identified using discriminant analysis of principal components between populations separated by less than five kilometers at the same elevations. There was no evidence for historical population decline, yet there was signal for recent demographic contractions, possibly resulting from environmental stochasticity. Correlative analyses revealed an association between patterns of genetic variation and annual heat-to-moisture ratio, mean annual precipitation, precipitation as snow and mean maximum summer temperature. Changes in climatic regimes forecasted for the region may thus potentially increase the rate of population extirpation by further reducing dispersal between sites. Consequently, American pika may have to rely on local adaptations or phenotypic plasticity in order to survive predicted climate changes, although additional studies are required to investigate the evolutionary potential of this climate change sensitive species.

## Introduction

Anthropogenic activities are having an unprecedented impact on wild populations. Perhaps one of the largest challenges facing wildlife will be coping with climate change [1]. Altered selection regimes caused by increasing air and water temperatures will operate in the near future [2], however, some particularly sensitive species have already become extirpated due to contemporary temperature changes [3]. When faced with rapidly changing environmental conditions, species must adapt, disperse or disappear [4]. While it has been suggested that most species will shift their geographical ranges rather than adapt *in situ* [5], factors such as habitat fragmentation may act synergistically to impair species' dispersal to more favorable conditions [6]. Range shifts may be particularly challenging for species with limited dispersal capacity or those with highly specialized habitat requirements [7]. Consequently, there is value in investigating the connectivity of populations of species inhabiting different environments in order to evaluate dispersal as a potential strategy for persistence in the face of predicted climate change.

Species that have successfully established populations along a continuous range of elevations may represent ideal systems in which to study the synergistic effects of genetic drift, inbreeding and natural selection on the extent and distribution of genetic variation as environmental conditions (e.g. temperature) change rapidly over short distances [8]. In a spatial context, altitudinal gradients can thus be used as surrogates for the expected temporal changes in selection pressures caused by climate change [1].

The American pika (*Ochotona princeps*) is a small lagomorph discontinuously distributed in mountainous areas throughout western North America from central British Columbia (BC) and Alberta, Canada, south to the Sierra Nevada in California and east to New Mexico, USA. Pikas are restricted to talus slopes and broken rock debris in proximity to meadows that provide their food [9]. The fragmented nature of their habitats has propelled *O. princeps* to the position of a model mammalian species for studies of metapopulation dynamics, island biogeography and source-sink dynamics [10]. In recent years, pikas have also gained notoriety as a model system for testing extinction dynamics in the face of climate change [3]. In that vein, pikas are considered harbingers of global warming, predicted by some to constitute the first mammalian species to go extinct due to the direct effects of climate change [11].

Recent studies have examined American pika phylogeography in the context of climate change since the late Pleistocene [12]. Additionally, DNA fingerprinting has been applied to shed light on mating behaviour and dispersal in this species [10], [13]. These studies identified that habitat fragmentation does not appear to prevent dispersal in pikas at small spatial scales (<10 km) in continuous or semi-continuous talus habitat. Yet investigations at larger spatial scales may reveal different patterns, especially those spanning rapidly changing environmental conditions. Given the above, *O. princeps* may represent an excellent system in which to study the relative importance of stochastic (genetic drift) versus deterministic (natural selection) processes in shaping genetic variation within a climate change-sensitive species.

In the present study, we begin to address these questions by sampling *O. princeps* populations found along three elevation gradients in the central Coast Mountains of British Columbia, Canada, ranging from sea level to greater than 1500 m. Specific objectives include: 1) investigating the extent and distribution of genetic variation within and among populations; 2) quantifying gene flow among populations along continuous, altitudinally distributed transects as well as disjunctly among transects; 3) evaluating the degree to which patterns of genetic variation correlate with observed environmental variation, and 4) reconstructing population demographic histories.

## Methods

### Study Site

**Figure 1:**
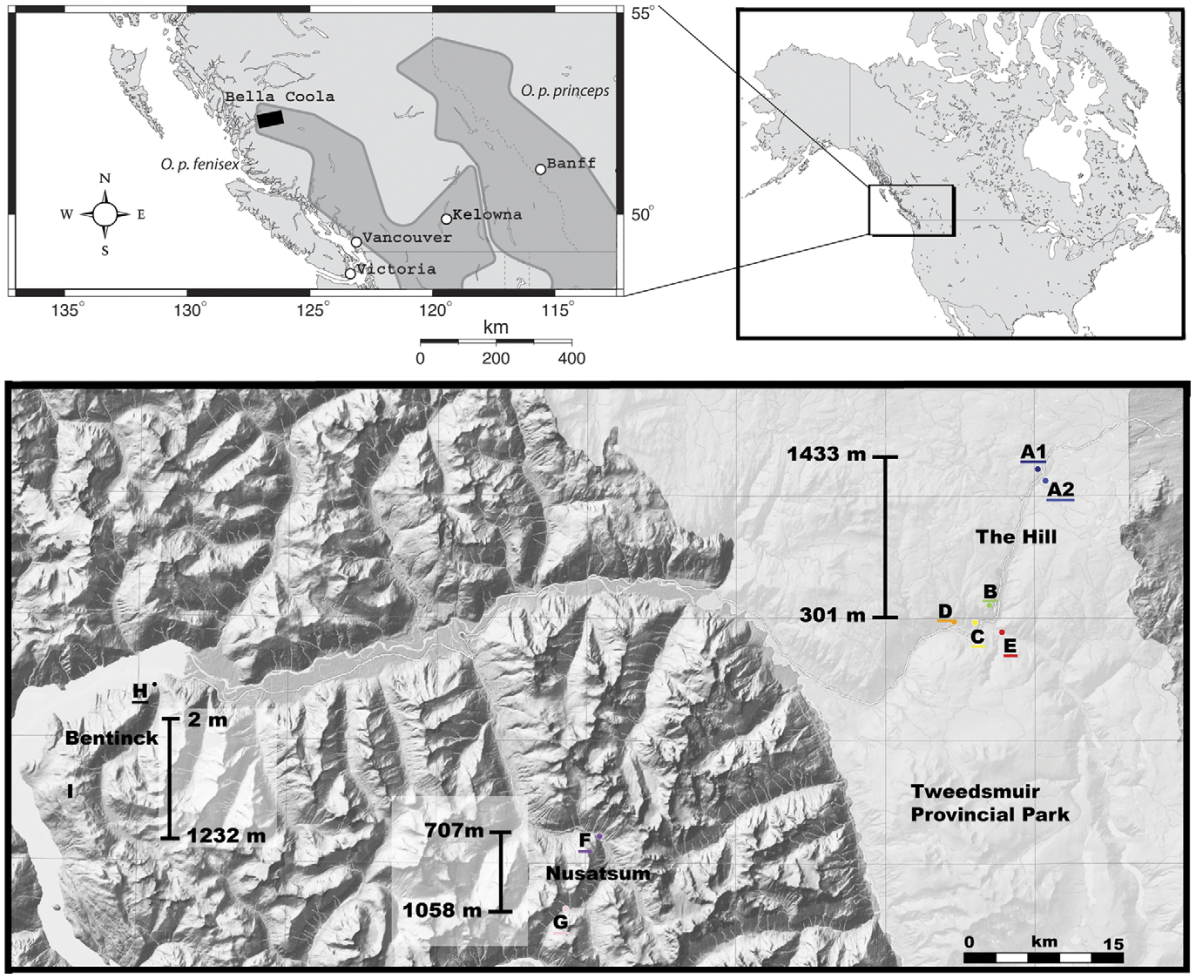
Map of the study area in the Bella Coola Valley, British Columbia, Canada, with the ten sampling sites located along three altitudinal gradients (lowest and highest elevations indicated): the Hill, Nusatsum and Bentinck from east to west. The light shading indicates Tweedsmuir South Provincial Park. The inset on the top left indicates the distribution of O. princeps subspecies.

This study was carried out in the Bella Coola Valley, BC (Fig. 1). This area was initially chosen because historical records show the presence of *O. princeps* from sea level to tree line and the presence of an extensive network of roads connecting the bottom of the valley to higher elevations. In this study area, talus slopes are highly fragmented and scattered throughout the landscape at relatively larger distances from each other (1 km to >70 km; Table S1). American pikas were sampled from August 2008 to September 2010 at 10 sites along three elevation gradients ranging from sea level to 1500 m using recently developed noninvasive hair snares (Fig. 1) [14], [15], and following the animal care protocol from the University of British Columbia (Certificate number: A07-0126). Pikas at site *A1* were potentially extirpated in the late summer of 2009 due to a forest fire that burned through the area, but the site was re-occupied as of summer 2011 (Michael Russello, personal communication). Samples were collected in sites *B*, *C*, *D* and *E* in 2008, 2009 and 2010, from site *A1* in 2008 and 2009, and from sites *A2, F, G, H and I* in 2010. A total of 288 geo-referenced individual hair snares were set up across these ten sites (Fig. 1) and samples were brought back to the laboratory for subsequent DNA extraction and PCR amplification.

### DNA Isolation, PCR Amplification and Genotyping

We previously determined that DNA extracted from 15–20 hairs per sample yielded DNA of sufficient quality and quantity for downstream assays [15]. Given these findings, we isolated DNA from 206 individual samples with sufficient starting material. DNA was extracted using the DNA IQ^TM^ Tissue and Hair Extraction Kit (Promega, Madison, WI, USA) and a modified version of the manufacturer's protocol as demonstrated by Henry *et al.* [14].

Isolated DNA was used as template for PCR amplification of 28 nuclear microsatellite loci previously described for the American pika (Table S2; Peacock et al., unpublished) [16]. Initially we used a representative sample of 16 individuals distributed throughout our study area to assess amplification success and polymorphism of all microsatellite loci (Table S2). Based on results from the pilot analysis, ten polymorphic loci were retained and screened on the remainder of the sampled individuals (see Results). All PCRs were performed using a Veriti® thermal cycler (Applied Biosystems, Foster City, CA, USA) in a 12.5 µL volume containing: 1–20 ng DNA, 0.5 µM of labelled M13, 0.5 µM reverse primer and 0.05 µM forward primer, 10 mM Tris-HCl (pH 8.3), 50 mM KCl, 1.5 mM MgCl_2_, 200 μM dNTPs, 10 µg bovine serum albumin (BSA; New England Biolabs, Ipswich, MA, USA) and 0.5 U AmpliTaq Gold® DNA polymerase (Applied Biosystems). All forward primers were 5′-tailed with an M13 sequence [5′-TCCCAGTCACGACGT -3′] to facilitate automated genotyping. Specifically, the M13-tailed forward primer was used in combination with an M13 primer of the same sequence 5′-labeled with one of four fluorescent dyes (6-FAM, VIC, NED, PET; Applied Biosystems; Table S2), effectively incorporating the fluorescent label into the resulting PCR amplicon [17].

Cycling parameters were optimised using a touchdown cycling program (10 min at 95°C, 40 cycles at 95°C for 30 s, 30 s annealing, and 45 s at 72°C, followed by a final step at 72°C for 10 min; Table S2). The annealing temperature decreased by 1°C per cycle from 60 to 55°C until reaching the sixth cycle, at which point the 34 remaining cycles continued at 55°C. Two of the markers produced stuttering using this touch down protocol and thus were amplified using a program that consisted of the same PCR cycling conditions except the annealing temperature was kept constant at 50°C for 40 cycles (Table S2). PCR products were multiloaded and run on an ABI 3130XL genetic analyser (Applied Biosystems) with GeneScan™ 500 LIZ® size standard and genotypes were called using Genemapper 4.0 (Applied Biosystems). Two people (Philippe Henry and Zijian Sim) called alleles independently and incongruent calls were repeated from the PCR stage.

### Quality Control and Genetic Variation

Since our sampling strategy involved the use of a non-conventional DNA source, we applied a multi-tube approach in which each PCR was repeated at least twice for heterozygote genotypes and at least three times in order to confirm homozygotes. Genotyping errors were quantified and consensus genotypes were obtained from repeated genotypes (up to five repeats per individual for 25% of samples) using Pedant 1.0 [18]. Additional tests for stuttering and allelic dropout were undertaken using Microchecker 2.2.3 [19]. Incidence of repeated genotypes were identified using GenAlex 6.4.1 [20] and duplicated multilocus genotypes were removed. Deviations from Hardy-Weinberg equilibrium (HWE) as well as linkage disequilibrium (LD) within sampled sites were assessed using Arlequin 3.5.1.2 [21] with the following settings: 1,000,000 steps in MCMC, and 100,000 dememorisation steps. Significance of deviations from HWE and LD were determined after sequential Bonferroni correction for multiple comparisons.

Within population genetic variation was quantified using observed and expected heterozygosity (*H_O_, H_E_*) and the number of alleles (*Na*) calculated in Arlequin 3.5.1.2 [21]. In addition, we used a measure of allelic richness (*A_R_*) based on a rarefaction index accounting for differences in sample sizes as implemented in Fstat 2.94 [22]. This index of allelic richness scales estimates based on the number of alleles observed in the sample with the lowest number of individual. The inbreeding coefficient (*F_IS_*) was also calculated for each site and its deviation from zero was assessed using 100,000 permutations between loci using Fstat 2.94 [22].

### Population Genetic Structure

Genotypic differentiation between pikas in different study sites was tested using a log-likelihood G-test not assuming HWE within samples using Fstat 2.94 [22] and based on 100,000 permutations. In this case, the software tests for the significance of population differentiation by permuting genotypes among sites, and comparing the random estimate to the observed. In order to shed additional light on underlying population genetic structure, we used discriminant analysis of principle components (DAPC) [23]. This model-free approach extracts information from genetic data by transforming the genotypes into uncorrelated components using principal components analysis (PCA). A discriminant analysis is then applied to a number of principal components retained by the user in order to maximize the among-population variation and minimize the variation within predefined groups. The fact that these methods lack underlying assumptions such as HWE and LD make them applicable to a wide range of situations where such assumptions are not met that may preclude the use of more conventional approaches such as Structure [24]. Recent studies that used both DAPC and Structure to shed light on population genetic structure provided similar outcomes based on the two methods [25], [26]. We ran the DAPC analyses using the R package Adegenet 1.3-1 [27]. Specifically, we applied the *a.score* and *a.optim.score* functions to identify the optimal number of principal components to be retained. We used 16 principal components representing 88% of the total genetic information and using sites as *a priori* populations for the DAPC.

To estimate rates and direction of recent migration events between each sample site, we used the Bayesian method implemented in BayesAss+ 1.3 [28] with the following parameters: 2 independent chains of 3,000,000 iterations with sampling every 2000 iterations, 999,999 burnin and delta values of 0.15 for *p*, *m* and *F* (corresponding to allele frequency, migration rate and inbreeding coefficient, respectively). This method makes use of gametic disequilibrium information in a Bayesian inferential framework to estimate recent (last two generations) migration rates from one population into another. This approach has one major assumption, namely that the loci used are in linkage equilibrium, but it does not require that populations are in HWE.

To test for associations between local environmental conditions and observed population genetic structure, we conducted Mantel tests to characterize the association between pairwise *Fst* and the environmental variables using *mantel.rtest* function of the R package ade4 [29] with 9,999 permutations. Our framework included the comparison of genetic differentiation (*Fst*) with population geographical isolation [30], elevation and five other environmental variables (mean annual temperature, mean annual precipitation, precipitation as snow, mean maximum summer temperature and heat-to-moisture ratio) calculated for each site using climateBC 3.1 [31]. This software downscales and interpolates PRISM 1961–1990 monthly normal data (2.5×2.5 arcmin) into 100 m×100 m resolution and outputs a number of measured and derived variables. Initially, we targeted the 39 annual and seasonal environmental variables available through climateBC. In order to remove redundant information from this large number of variables, we performed a principal component analysis (PCA) and calculated correlation coefficients between each pair of variables using the R packages ade4TkGUI [32] and Rcmdr [33] respectively. Variables were considered as redundant if they produced a correlation coefficient higher than 0.8, as suggested by Manel *et al.* [34], in which case the variables that were least biologically relevant (e.g. derived variables or variables that *a priori* do not affect the species) were removed from further analyses.

Additionally, we applied a hierarchical Bayesian method that estimates local *Fst* values and relates them to environmental variables of interest as implemented in Geste 2 [30]. Generalized linear models were run using all seven factors and resulting in a total of 2^7^ (128) models. The method evaluates the posterior probabilities of each factor and their combinations in shaping the observed population genetic structure using reversible jump Markov chain Monte Carlo (MCMC). For example, the model that compares only genetic differentiation and geographic isolation can be viewed as a null model for isolation by distance, while other models can incorporate more complex scenarios. We followed the approach of Gaggiotti *et al.* [35] in that we first ran Geste using all seven factors and then performed a second round of analyses using the three factors with the highest cumulative posterior probabilities from the first round of analyses.

### Demographic history

Genetic signatures of demographic contraction were assessed within each site using three different approaches: 1) the heterozygote excess test and 2) the mode-shift test, both implemented in the software package Bottleneck 1.2.02 [36], as well as 3) the *M*-ratio test using M_p_val.exe and critical_M.exe [37]. For the heterozygote excess test, significance was assessed using 100,000 iterations with the Wilcoxon sign-rank test and under a Two Phase Model (TPM) consisting of 10% multi-state change and a variance among multiple steps of 12 as recommended by Piry *et al.* [36]. For the *M*-ratio test, we used a TPM mutation model with 10% multi-state change, assuming a marker mutation rate *μ* of 5×10^−4^ and a pre-bottleneck *N_e_* ranging from 500 to 12,500 resulting in a value of *θ* (4 *N_e_* μ) ranging from 1 to 25 and 3.5 bases steps for multi-step mutations. We used both approaches since the heterozygosity excess and mode shift test have been suggested to reflect recent demographic contractions while *M*-ratio reflect historical contractions [38].

## Results

### Quality Control and Genetic Variation

Of the 28 microsatellite loci screened, six markers failed to amplify and 12 markers were monomorphic (Tables S1). Ten markers were polymorphic and used to genotype the 206 hair samples. The overall dataset contained 2.8% missing data, ranging from 0% (*Ocp13*) to 7.7% (*Ocp12*).

The multi-locus probably of identity for this suite of ten microsatellite loci was 8.3×10^−6^. Of the 206 hair samples genotyped, 38 pairs harbored identical multi-locus genotypes. We removed one sample from each pair, resulting in a final dataset containing 168 individuals. The mean number of individuals sampled per site was 16.2, ranging from 5 (*H*) to 32 (*D*) (Table 1).

**Table 1:** Site-specific information including site names, transect, geographical location, elevation, sample size (N), observed (Ho) and expected (He) heterozygosites, number of alleles (Na), allelic richness (Ar), and within-site inbreeding coefficient (Fis), values in brackets indicate Standard Error.

Site	Transect	Latitude	Longitude	Elevation (m)	N	Ho	He	Na	Ar	Fis
A1	Hill	N52° 18' 36"	W125° 29' 47"	1433	15	0.51 (0.23)	0.58 (0.19)	3.2 (1.0)	2.8 (0.84)	0.10
A2	Hill	N52° 18' 26"	W125° 29' 34"	1338	6	0.40 (0.31)	0.61 (0.13)	3.1 (1.3)	2.6 (1.29)	0.38
B	Hill	N52° 15' 9"	W125° 31' 39"	793	17	0.63 (0.17)	0.67 (0.11)	4.4 (1.7)	3.5 (0.82)	*0.06*
C	Hill	N52° 14' 56"	W125° 32' 14"	362	26	0.52 (0.15)	0.68 (0.09)	4.6 (1.2)	3.5 (0.64)	0.23
D	Hill	N52° 14' 49"	W125° 33' 15"	301	32	0.56 (0.14)	0.69 (0.08)	5.4 (1.4)	3.7 (0.70)	0.20
E	Hill	N52° 14' 39"	W125° 31' 14"	329	21	0.55 (0.18)	0.60 (0.09)	5.0 (1.2)	3.2 (0.42)	0.08
F	Nusatsum	N52° 9' 37"	W126° 11' 29"	707	10	0.38 (0.19)	0.56 (0.14)	3.2 (0.6)	2.9 (0.48)	0.34
G	Nusatsum	N52° 7' 46"	W126° 13' 4"	1058	30	0.46 (0.16)	0.64 (0.14)	4.3 (1.1)	3.3 (0.63)	0.28
H	Bentinck	N52° 13' 21"	W126° 29' 22"	2	5	0.45 (0.18)	0.59 (0.12)	2.6 (0.7)	2.3 (0.95)	0.26
I	Bentinck	N52° 10' 22"	W126° 32' 5"	1282	6	0.28 (0.19)	0.36 (0.17)	2.3 (0.5)	1.7 (0.73)	0.25

After correcting for multiple tests, eight out of ten microsatellite loci were found to deviate from Hardy-Weinberg expectations (*HWE*) in at least one of the ten sampled sites (Table S3). *Ocp15* showed the highest evidence for violation of this assumption, deviating from *HWE* in five of ten sites. The other loci that deviated from *HWE* did so at only one or two sites (Table S3). Since we found no systematic deviation of *HWE* in all sites, all loci were retained for further analyses, but for tests that assumed loci were in *HWE*, we repeated the analyses without the loci that violated this assumption.

Linkage disequilibrium (*LD*) occurred between four pairs of loci, yet this significant linkage was never observed at all sites (Table S4). The most frequent pair of loci exhibiting deviation from linkage equilibrium (*Ocp12*/*Ocp23*) occurred in five of 10 sites (*C, D, E, F, G*; Table S4). The other three instances of *LD* were specific to only one (*D*: *Ocp2/Ocp11*; *E*: *Ocp15/Ocp22*) or two sites (*A, D*: *Ocp2/Ocp6*; Table S4). As above, when the underlying assumptions of the tests required that loci be at linkage equilibrium, one locus from each linked pair was omitted from these calculations. Furthermore, no evidence for stuttering was detected and allelic dropout and false allele rates were estimated based on repeated genotypes to be as low as 0.002 and 0.0015 respectively, resulting in a total error rate of 0.0035.

Our samples harboured 73 alleles at ten microsatellite loci, ranging from four to 11 alleles per locus. The three sites with the lowest sample sizes contained monomorphic loci: *A2* (*Ocp12* and *Ocp23*), *H* (*Ocp22* and *Ocp 7*) and *I* (*Ocp12*, *Ocp22*, *Ocp23* and *Ocp7*) while all other sites were polymorphic at all ten loci (Table S5). Within population genetic variation was relatively low with a mean observed heterozygosity of 0.47 (SD =0.1), ranging from 0.63 in site *B* to 0.28 in site *I* (Table 1). A similar pattern was revealed for allelic richness with a mean *Ar* of 2.95 (SD =0.66) with values ranging from 3.7 in site *D* to 1.7 in site *I*. Within-site inbreeding coefficients (*Fis*) were always positive and differed significantly from zero in all instances except one (site *B*; Table 1).

The amount of genetic variation found in the *O. princeps* samples from the Bella Coola Valley was lower, on average, than that previously reported in other studies of *Ochotona spp.* that employed an overlapping subset of the microsatellite markers used here [16], [39], [40], [41]. Although these studies only incorporated four and three out of the ten loci we used [*Ocp2*, *Ocp6*, *Ocp7* and *Ocp9* in 16,39,41] and [*Ocp2*, *Ocp7* and *Ocp9* in 40], meaningful comparisons are still possible. After removing the non-overlapping loci, mean observed heterozygosity was 0.45 (SD =0.19). This value is substantially lower than those reported in previous studies of *O. princeps* from Montana, California and Nevada 0.65, SD =0.14 [16]; 0.64, SD =0.15 [41]. The levels of observed heterozygosity reported here are also lower than those found in previous studies of congenerics, including 0.64 (SD =0.19) from Yukon *O. collaris* [39], and 0.72 (SD =0.25) from *O. curzoniae* sampled from the Qinghai-Xizang plateau, China [40]. Additionally, the allelic richness we observed (*Ar* =3.3; SD =0.33) was also lower than that found in *O. princeps* populations in Nevada, USA [*Ar* =4.8; SD =0.99; [41].

### Population Genetic Structure

Log-likelihood tests of population differentiation between sites were all significant, except in the pairwise comparisons that involved the sites with the smallest sample sizes (*A2*-*H*, *A2*-*I*, *H*-*I*). While average *Fst* values for most sites were centred around 0.20 (0.17 – 0.26), the high elevation site from the Bentinck transect (*I*) displayed an unusually high *Fst* value of 0.36 (Table 2).

**Table 2:** Pairwise population differentiation indices.

	A1	A2	B	C	D	E	F	G	H	I
A1	-	*	*	*	*	*	*	*	*	*
A2	0.20	-	*	*	*	*	*	*	NS	NS
B	0.14	0.22	-	*	*	*	*	*	*	*
C	0.14	0.16	0.11	-	*	*	*	*	*	*
D	0.15	0.22	0.11	0.05	-	*	*	*	*	*
E	0.19	0.19	0.17	0.15	0.14	-	*	*	*	*
F	0.25	0.21	0.14	0.16	0.20	0.23	-	*	*	*
G	0.17	0.20	0.12	0.18	0.16	0.19	0.10	-		*
H	0.26	0.29	0.22	0.22	0.25	0.29	0.25	0.19	-	NS
I	0.40	0.43	0.35	0.35	0.34	0.32	0.38	0.30	0.38	-

*FST* values are represented below the diagonal. Asterisks above the diagonal represent a significant differentiation based on log-likelihood G-tests (Goudet, 1996)

**Figure 2:**
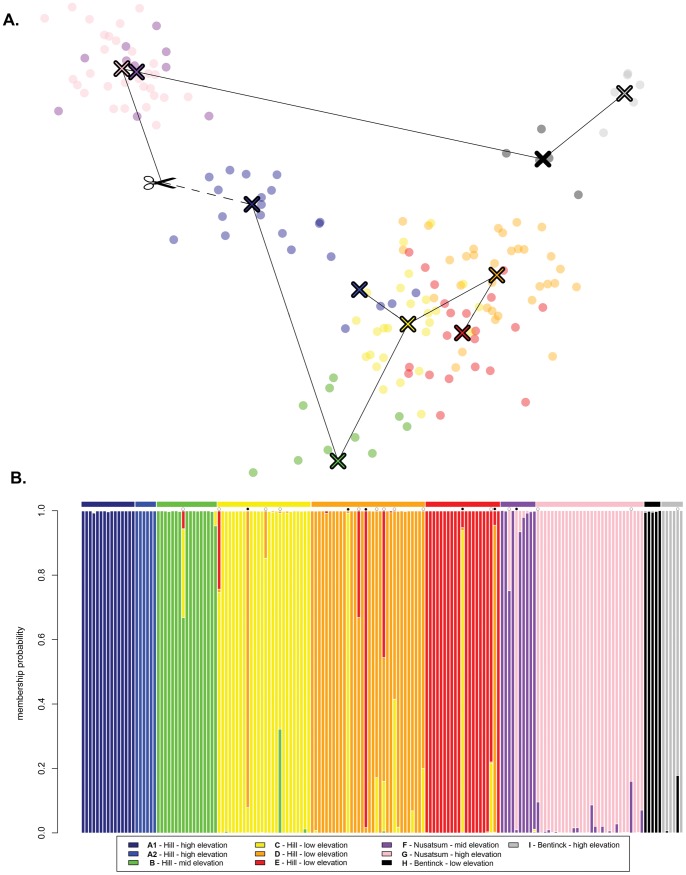
Population structure in pikas from the Bella Coola Valley. A. Scatter plot from the Discriminant Analysis of Principal Components (DAPC) [23] with a minimum spanning tree based on the squared distance between populations, showing the regional cohesion of sample sites into clusters that represent the three elevation gradients sampled. The centre of each group is indicated by a cross of the respective colour. Note that the distance between the Hill and the rest of the samples is not to scale (dashed line). B. Plot of membership probabilities, where each column represents an individual. Admixed individuals are defined as individuals that had less than 90% probability of membership to the site in which it was sampled and denoted by an open circle. Migrant individuals are denoted by full circles.

The DAPC analysis grouped the sites into clusters corresponding to the three elevational transects (Fig. 2a). The Hill samples were separated from the Bentinck sites by the first axis of the DAPC and from the Nusatsum samples by the second axis, while the Nusatsum samples were separated from the Bentinck samples by the first axis only (Fig. 2a). Evidence of admixture between populations was found within all three transects (Fig. 2b), with the presence of potential first generation migrants identified in the low elevation sites from the Hill (5 individuals with membership probabilities higher than 90% [42] from *C*, *D* and *E*; black circles in Fig. 2b), and an additional individual in Nusatsum. Additionally, the presence of admixed individuals (individuals with membership probabilities lower than 90% [42]) was detected in *B*, *C*, *D*, *E*, *F*, *G* and *I* (open circles in Fig. 2b). The highest frequency of admixture occurred between the low elevation populations from the Hill, which are in close geographical proximity (less than five kilometres). There was no evidence of admixture between low and high elevation sites across all possible comparisons.

Estimates of recent migration rates (*m*; the proportion of migrant individuals per populations per generation) yielded results that were mostly consistent with observed patterns of population differentiation, in that mean migration rates were low in all cases, (0.001 to 0.07; Table 3; Table S6). These values are lower than the suggested migration rate at which two populations are considered to exchange sufficient migrants to influence each other's dynamics [m=0.1; 43], yet the affinities highlighted by the DAPC approach are also detected, especially between the low elevation sites from the Hill transect where values of migration rates approached this threshold. Additionally, on several instances the upper limits of the confidence intervals approach or are above 0.1, consistent with the DAPC results. It is also interesting to note that migration rates for site A2 are one order of magnitude higher than all other populations. The low sample size at this site are likely responsible for these larger estimates.

**Table 3:** Mean estimates of the distribution of recent migration rates (*m*) calculated using Bayesass+ [28], given as the proportion of migrant individuals per population per generations.

	A1	A2	B	C	D	E	F	G	H	I
A1	**0.971**	0.074	0.004	0.004	0.005	0.011	0.005	0.001	0.009	0.004
A2	0.003	**0.71**	0.003	0.003	0.003	0.004	0.006	0.001	0.008	0.005
B	0.003	0.015	**0.965**	0.047	0.005	0.008	0.006	0.002	0.011	0.005
C	0.003	0.043	0.004	**0.899**	0.015	0.017	0.006	0.001	0.008	0.004
D	0.003	0.015	0.005	0.017	**0.946**	0.006	0.005	0.002	0.008	0.004
E	0.004	0.017	0.004	0.003	0.013	**0.914**	0.005	0.001	0.008	0.005
F	0.003	0.021	0.004	0.011	0.003	0.007	**0.933**	0.002	0.009	0.005
G	0.004	0.046	0.004	0.007	0.004	0.009	0.015	**0.986**	0.009	0.005
H	0.003	0.016	0.004	0.005	0.003	0.01	0.006	0.002	**0.918**	0.005
I	0.003	0.043	0.004	0.004	0.002	0.016	0.011	0.001	0.014	**0.959**

Columns represent the incoming migration rates and rows represent the outgoing migration rates. Bold values represent the proportion of non-migrant individuals in a population.

The results of the Mantel tests indicated that geographical isolation is the environmental variable that correlates most strongly with genetic differentiation (r^2^=0.75, p=0.003), followed by heat to moisture ratio (r2=0.37, p=0.01), precipitation as snow (r2=0.37, p=0.05), mean annual precipitation (r2=0.31, p=0.04) and summer mean maximum temperatures (r2=0.19, p=0.05). All other variables were not significantly correlated with genetic differentiation. Additionally, Bayesian model testing using all seven factors identified mean maximum summer temperature as the variable with the highest cumulative probability (*Pr=*0.5) in explaining the observed population genetic structure, followed by geographic isolation (*Pr=*0.497) and mean minimum winter temperature (*Pr=*0.493). The model with the highest probability (*Pr=*0.01) contained all three variables listed above. The second round of MCMC containing only these three factors identified that the model with the highest posterior probability contained a constant term (*a0=*−2.13) and mean maximum summer temperature only (*Pr=*0.134). This latter model resulted in a positive regression coefficient (*a_1_*=0.205), suggesting that genetic differentiation increases with increasing temperature. Yet, a high proportion of the variance remains unexplained by this model as illustrated by a large value of model fit coefficient (*σ^2^*=1.13), indicating that Geste failed to detect the true model [30].

### Demographic history

Signatures of recent demographic contraction were evidenced in half the sites as they displayed a significant excess heterozygosity relative to the number of alleles expected at mutation-drift equilibrium (Table S7). Four of these five sites (*A1*, *C*, *G* and *H*) also displayed shifted allele frequency distributions, another indication of recent demographic contraction. These results were not affected when loci that deviated from *HWE* expectations were excluded from the analyses.

Conversely, the *M*-ratios were always larger than the critical *M* value simulated for a stable population with the same number of individuals and loci used here, suggesting historically stable populations. The outcome of the *M*-ratio tests remained unchanged when *θ* was varied from 1 to 25.

## Discussion

### Quality Control and Genetic Variation

In the present study, we used a noninvasive sampling approach that enabled us to effectively collect population-level samples of the American pika. Following appropriate quality control, our final microsatellite dataset presented a 0.35% error rate corresponding to 0.2% allele dropout (seven false homozygotes out of 3360 alleles) and 0.15% of false alleles (five false heterozygotes out of 3360 alleles), values that are towards the low end of reported genotyping errors for high quality starting material such as plucked hair [44].

Given the extent of quality control used in our study, the observed deviations from *HWE* at several loci in some of our sites are most likely biologically meaningful, associated with pika life history rather than by genotyping errors [45]. Indeed, *O. princeps* populations are usually small (at most 10 individuals per ha) [41] and exhibit non-random mating [13]. Additionally, inbreeding coefficients were significantly above zero in nine out of ten of our study sites, indicating that the hypothesis of random mating should be rejected. These results are in contrast with those observed in *O. princeps* populations from the centre of their range, where other studies did not find evidence of inbreeding [13], [41], [46]. Other life history characteristics that may have contributed to high within-site inbreeding coefficients and deviations from *HWE* are the potential for overlapping generations, post-glacial colonization, limited dispersal capacities (see below) and metapopulation structure. Regarding the latter, we directly observed the impacts of environmental stochasticity over the course of this study, as illustrated by the potential extirpation of one of the high elevation sites on the Hill transect (*A1*) due to a 2010 forest fire that was subsequently re-occupied in 2011. Further research would be required, however, in order to definitively determine whether pikas observed at *A1* in 2011 were the result of re-emergence or recolonization from a neighbouring source population that was not sampled in the current study.

The low level of genetic variation recovered in our samples may be explained by one or a combination of the following: 1) relatively small population sizes; 2) measurable levels of inbreeding; and 3) location of the Bella Coola Valley at the northwestern tip of the American pika distribution. Based on this latter consideration, lower levels of genetic variation observed in Bella Coola Valley *O. princeps* populations may be a result of a more recent post-glacial expansion from southerly refugia compared to core populations in the USA [47]. These results are also consistent with population genetic theory that predicts that populations located at range margins are more isolated from sources of immigrants and are more susceptible to the stochastic processes and bottlenecks that lead to depleted neutral genetic variation. In practical terms, these observed low levels of genetic variability may represent an impediment to the evolutionary potential of our study populations [7], although see [48].

### Population Genetic Structure, limited dispersal and the effect of environmental conditions

The marked population genetic structure revealed in this system is not surprising given the life history characteristics of pikas (e.g. limited dispersal abilities, high mortality of dispersing individuals). All sites displayed significant differentiation except for the pairwise comparisons of the three sites that contained the fewest samples (*A2*, *H*, *I*; n<10). As the *Fst* values for these sites were still very high, and in the case of site *I*, the highest observed in our samples, it is quite likely that the lack of significance for these comparisons is an artefact of small sample sizes. Consequently, each sampled site likely represents an independent unit characterized by a unique allele frequency distribution. Yet, it has been suggested that pikas are able to disperse to neighbouring talus patches located up to three kilometres apart [41], [46]. This pattern was also found here, especially between sites at similar elevations and/or those that are less than three km apart (*B* – *C*:1.3 km, *C* – *D*:2 km, *D – E*:2 km), as well as between sites *C* and *E* separated by five kilometres. It is noteworthy that site *A1* and *A2*, although separated by less than a kilometre did not display evidence for any exchange of migrants, which is most likely due to the local topography that contains a deep valley crossed by a torrent, thus producing an effective barrier to dispersal. Estimates of migration rates corroborated this finding, although the fact that this approach relies on an island model of population structure may bias estimates of gene flow downwards [36]. In our case, where populations potentially exhibit a hierarchical stepping stone meta-population structure, this approach may thus not be completely appropriate, and these results should be interpreted with caution.

Although low levels of gene flow were generally detected between sites, we did uncover regional cohesion at the level of the transect (Fig. 2a). These results suggest that all sites within a transect have a common origin, with some exchange of migrants. On a broader scale, the low elevation of the Bella Coola Valley floor (sea level to 300m) may constitute a major barrier to gene flow between transects over the distances studied here (35 to 70 km). Yet, suitable pika habitat does exist between transects, leading to the possibility that recovered patterns of differentiation may be influence by our sampling scheme. However, given our findings of fine-scale structure (complete isolation of populations 10km apart), the lack of complete sampling between transects likely would only affect inferences regarding the magnitude, not the presence, of significant differentiation.

Along the same line, we identified that geographical isolation is the one predictor that best explains the underlying population genetic structure, as demonstrated by the highest regression coefficient in Mantel tests, thus indicating a significant pattern of isolation by distance. The outcomes of the Bayesian model testing did not corroborate these results, yet the fact that we included only ten populations may be prohibitive as the software Geste may not perform ideally when samples of less than 20 populations are available [30].

Previous studies have shown that pikas are at high risk of hyperthermia if exposed to ambient temperatures above 27°C and behaviourally thermoregulate by seeking shelter in their rocky habitat where temperatures remain cooler [49]. Dispersal thus represents a major source of mortality due, in part, to the inability for thermoregulation during attempted migrations, which may differ at varying elevations. Indeed, we found that temperatures differed significantly along one of our elevation gradients (the Hill; Fig. 1), varying up to six degree Celsius from high to low elevation sites (mean temperatures =14°C to 20°C, maximum temperatures =20°C to 35°C) [50]. Moreover, this work quantified the insulation properties of talus habitat, finding that below talus temperatures were significantly lower than above talus temperatures throughout the afternoon, and were significantly warmer than above talus temperatures in the morning and night [50].

American pika population extirpations have been correlated with increasing rates of environmental change over the past decade within the southern part of their range [3]. In the present study, we detected evidence for recent population decline in half the sampled sites, found at different elevations. A similar pattern was observed by Merideth [41], where two of six populations displayed genetic signatures of population decline, but failed to detect evidence of historical contractions. In terms of potential processes leading to observed declines, correlative analyses found an association between patterns of genetic variation and the annual heat to moisture ratio, mean annual precipitation, precipitation as snow and mean maximum summer temperatures. Changes in temperature and precipitation regimes that are forecasted for the region in the near future may thus potentially increase the rate of population extirpation, by reducing the dispersal occurring between sites. Thus, limited thermal tolerance and restricted dispersal capacity may interact synergistically with future climate warming to inhibit recolonization of extirpated patches and reduce survival of resident animals, leading local populations into an extinction vortex.

### Conclusion

The present study revealed low levels of neutral genetic variation in *O. princeps* populations from the northern edge of their distribution. While the implications of such impoverished gene pools on the persistence of *O. princeps* are unknown, low levels of genetic variation may increase risks of extinction [51]. Additionally, evidence for restricted gene flow between local populations sampled along continuous altitudinal gradients suggest that, in the face of climate change, *O. princeps* may have to rely on local adaptations or phenotypic plasticity in order to survive the predicted magnitudes of environmental change. Future studies using non-neutral molecular markers are required, however, in order to shed light on the evolutionary potential of this climate change sensitive species.

## Supporting Information

Table S1 **Pairwise Euclidean geographical distances** (**m**) **between sample sites.**
(XLS)

Table S2 **Information on the microsatellite loci tested in the present study including locus name, Genbank numbers, length** (**for successful amplification**) **and PCR program** (**for loci retained in the present study**)**, the dye and multiload used. Loci with a single fragment length were monomorphic in our samples.**
(XLS)

Table S3 **Tests of Hardy Weinberg equilibrium** (***HWE***) **within each site for each loci used.** Black boxes indicate significant deviation from *HWE* per locus per site after corrections for multiple comparisons.
(XLS)

Table S4 **Test of linkage disequilibrium for each pair of loci calculated within sites.** Combined *p*-values are given below the diagonal, and sites at which the two loci were linked are given above the diagonal.
(XLS)

Table S5 **Observed** (***Ho***) **and expected** (***He***) **heterozygosities for each locus and each site.**
(XLS)

Table S6 **Mean estimates of the distribution of recent migration rates** (***m***) **calculated using Bayesass+** [**28**] **, given as the proportion of migrant individuals per population per generations.**
(XLS)

Table S7 **Results of the tests of demographic history for each site.**
(XLS)
